# Detection Method for Bolted Connection Looseness at Small Angles of Timber Structures based on Deep Learning

**DOI:** 10.3390/s21093106

**Published:** 2021-04-29

**Authors:** Yabin Yu, Ying Liu, Jiawei Chen, Dong Jiang, Zilong Zhuang, Xiaoli Wu

**Affiliations:** College of Mechanical and Electronic Engineering, Nanjing Forestry University, Nanjing 210037, China; yabinyu_new@163.com (Y.Y.); cjw@njfu.edu.cn (J.C.); jiangdong@njfu.edu.cn (D.J.); zzl0702@njfu.edu.cn (Z.Z.); wxl2002@njfu.edu.cn (X.W.)

**Keywords:** deep learning, machine vision, bolt looseness angle

## Abstract

Bolted connections are widely used in timber structures. Bolt looseness is one of the most important factors leading to structural failure. At present, most of the detection methods for bolt looseness do not achieve a good balance between cost and accuracy. In this paper, the detection method of small angle of bolt loosening in a timber structure is studied using deep learning and machine vision technology. Firstly, three schemes are designed, and the recognition targets are the nut’s own specification number, rectangular mark, and circular mark, respectively. The Single Shot MultiBox Detector (SSD) algorithm is adopted to train the image datasets. The scheme with the smallest identification angle error is the one identifying round objects, of which the identification angle error is 0.38°. Then, the identification accuracy was further improved, and the minimum recognition angle reached 1°. Finally, the looseness in a four-bolted connection and an eight-bolted connection are tested, confirming the feasibility of this method when applied on multi-bolted connection, and realizing a low operating costing and high accuracy.

## 1. Introduction

In terms of the advantages of reliability and convenience, bolted connections are widely applied in timber structures. Wood packaging cases are impacted by continuous vibrations load during transportation, which causes bolted connections to loosen. Thus, the connections within could fail on account of the continuous vibration loads; therefore, research on the detection methods of timber structure bolt connection loosening is of great significance.

The detection methods of bolt loosening are mainly divided into two methods—direct detection and indirect detection. The main issue with bolt looseness is the low axial force, which leads to thread slip in the touching areas. Thus, the two key parameters in the direct detection method are axial force and applied torque. Most of these methods were proposed a long time ago, but they are still used in various industries. Goodier and Sauer et al. [1,2] developed a direct tension indicating method. The axial force was measured with a tension-indicating washer designed for specific joints and with a unique purpose. With an increase in axial force, the gap between the features on the washer and the bolt (or nut) is decreased. The advantage of this method is convenience, while the drawback is that each washer is specifically designed for each different sample tested. Bickford adopted the strain gauge method to measure the axial force applied on blots [3]. With two strain gauges installed on the two sides of a bolt, once the torque was applied on the bolt, the axial force to the joint increased and the gauges measured the applied force continuously. This test is only used when both sides of the bolt are accessible. The torque control method is also utilized by researchers to control and investigate the looseness of a bolt. With this technique, an inspector used a torque wench to determine the torque of a bolt at time intervals to check that it did not decline. However, by excessively relying on manual work, these methods resulted in a low accuracy, and the online testing of structures is not possible [4]. Therefore, because of the drawbacks of the direct measurement methods mentioned above, various indirect measurement methods have been developed and adopted for examining bolted connection looseness.

The theory of indirect measurement is used to determine the looseness of the bolts indirectly based on different physical parameters. Huo et al. [5] used a pair of piezoceramic transducers for ultrasonic and wave generation and detection in order to test bolt looseness in bolted connections. This method obtains the focused signal peak amplitude during ultrasonic wave propagation through the bolted connection surface, which reveals the value of the applied preload. Based on this technique, Xu et al. [6] optimized the gathering approach of the ultrasonic signal to improve the accuracy and to expand the range of sensitivity. Zhao et al. [7] applied this method on looseness testing of bolted connections on a wood structure, and put forward the loss index of the preload of a bolt to describe the looseness of a bolted connection in wood structures. Zhang et al. [8] developed a bolt looseness detection method based on audio classification. They recorded and extracted the sound of a bolted connection under different preloads that were hit by a hammer and determined the looseness results using support vector machine (SVM) training and collecting. By improving the audio data acquisition process, Wang et al. [9] proposed a new vibration-acoustic modulation (VAM) method to detect the looseness of multi-bolt connections, which could also detect other types of damage to a certain extent. These detection methods can basically realize unmanned on-line monitoring [10], but the disadvantage of these methods is that different signals are collected by specific sensors; the environment for obtaining the characteristic data needs to be small [11]; and in the case of a large number of bolted connections, a large number of sensors will increase the cost and difficulty of monitoring.

In the indirect detection method, there is also a method based on image processing, which has the advantage of a low implementation cost. Park and Tuan-Cuong [12,13] developed an image-based bolt looseness detection method for a steel structure. They identified the rotation angle of each nut using the Hough transform algorithm, in order to describe the looseness of a bolt. The identification angles ranged from 0° to 60°. Cha [14] combined the Hough transform algorithm with SVM to identify the height of a bolt relative to the bolt looseness. This method was effective at excessive large damage to bolts, but it could not identify a small degree of bolt looseness. With the rapid development of deep learning technology, image identification and location methods based on convolutional neural networks (CNNs) have achieved a high accuracy [15]. Hiton and Alex [16] proposed the AlexNet algorithm, in which GPU (Graphics Processing Unit) is initially used for computing the acceleration; this resulted in increased speed in image identification. After this, various superior neural network algorithms based on deep learning were discovered, namely VGGnet [17], GoogLeNet [18], R-CNN [19], Fast R-CNN [20], Faster R-CNN [21], YOLO [22], and Single Shot MultiBox Detector (SSD) [23], all of which comprehensively cover the rate and accuracy of identification. Sun et al. [24] suggested a binocular visual bolt looseness detection method applied on running train’s key components. They identified the edges of a bolt cap and mounting surface in the localized regions using CNN and calculated the distance between them. The looseness could be determined according to this calculation. Thanh-Canh et al. [25] increased the identification rate by combining the Hough transform and R-CNN, but the testing angle still ranged from 0° to 60°. Zhao et al. [26] utilized the SSD algorithm to identify the rotation angel of a bolt head. It relatively simplified the processing, while increasing the identification angle to a range of 0° to 360°. Nevertheless, in this approach, the minimum of identification angel was lowered to 10° and the average angle error was 4.47%, which did not test the looseness in a multi-bolted connection. Zhang et al. [27] measured the bolt height using the Faster R-CNN algorithm to determine its looseness state, while it did not work well for identifying small amounts of looseness for bolted connections. In summary, adopting deep learning to examine bolted connection looseness has many proven advantages, including low-cost operation, identification of various features, and increasing the testing rate and accuracy with the development of the algorithm.

**Figure 1:**
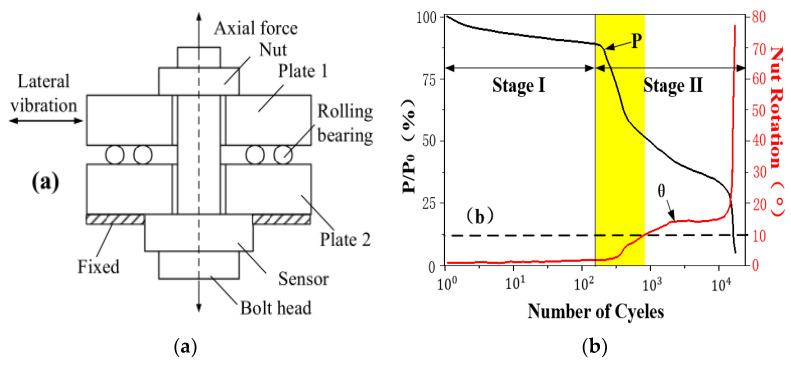
Experimental equipment of (**a**) bolt loosening and (**b**) curve of bolt looseness.

Up until now, most research on the mechanics of bolted connection looseness refer to the equipment designed by Junker [28]; Figure 1a. The testing results of the correlation between the bolt preload and rotation angle are given in Figure 1b [29], where P represents the preload and θ is the rotation angle of the nut. When no relative sliding exists between the bolt and nut, the preload curve stays relatively gentle in stage I, and the looseness angle is miniscule. As the bolt rotates, the preload curve declines quickly to stage II. Meanwhile, the rotation angle rapidly increases, which leads to the failure of the structure. From Figure 1b, it can be seen that the rotation angle is lower than 10° when the preload rapidly declines, and afterwards, when the preload declines to 25%, the rotation angle is around 20°. Therefore, it is efficient and feasible to predict and prevent bolted connection failure by testing bolt looseness at a small rotation angle.

Traditional image processing methods need tedious operations on images and require a certain expertise of a technician. The methods based on artificial image processing are easily influenced by different environments. Complex background, such as similar colors to the objects or too strong and too weak light causing visual fatigue, also interfere the visual identification from manual work that might lead to image processing incorrectness and failure. In the methods based on the deep learning, a technician only needs to provide a certain amount of training images and desired results to the computer itself to complete the training process and end up with a model generated. Users can obtain the results by inputting images, so it does not require high expertise. Furthermore, the self-training method can be applied in complicated environment. Before the deep learning algorithm comes out, for a visual algorithm, it can be divided into the following five steps: feature perception, image preprocessing, feature extraction, feature screening and reasoning prediction and recognition. Traditional machine vision recognition methods separate feature extraction from classifier design, and then combine them in application. However, the manual design of features of traditional visual algorithms calls for a lot of experience and requires a studier to have a special understanding of this specialty. The emergence of convolution neural network, as the core of deep learning, basically overcomes the problem of manual processing of image features. The more successful applications of deep learning in machine vision include face recognition, image question answering, object detection and object tracking. Based on the object detection technology in the field of deep learning machine vision, this paper studied the detection method of small-angle loosening of timber structure bolts. It covers the following three schemes: scheme A, to identify a nut and the specification pattern on it; scheme B, to identify the round feature on the bottom of a bolt bottom and the rectangle on a nut; and scheme C, to identify the round features on a bolt and nut. In each scheme, the same number of images were collected and trained with an SSD network to identify the object, and the coordinates were generated at the center of each object. Then, the bolt looseness angle was calculated according to the given coordinates. The results from the three schemes were compared and verified so as to pick the most efficient and accurate one. To further improve the accuracy, tests at angles less than 10° were carried out in order to find out the smallest identification angle. After that, the multi-bolted connections were also tested to examine the efficiency of this method. This study provided references for testing bolted connection states of timber structures in the early stage.

## 2. Materials and Methods

### 2.1. Image Collection and Calculation of Looseness Angle

**Figure 2:**
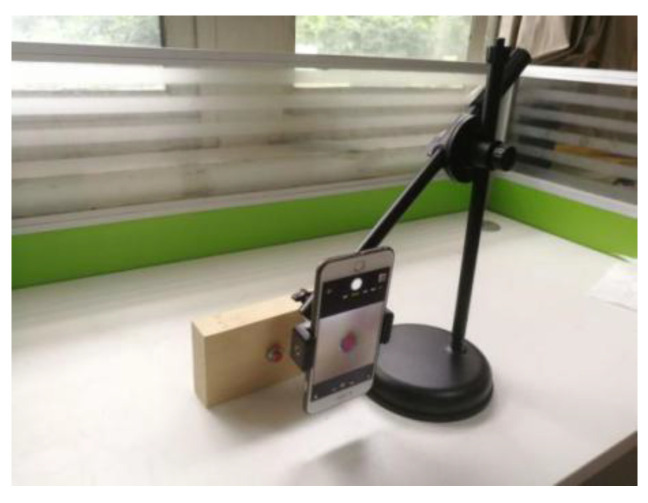
Setup of image collection.

**Figure 3:**
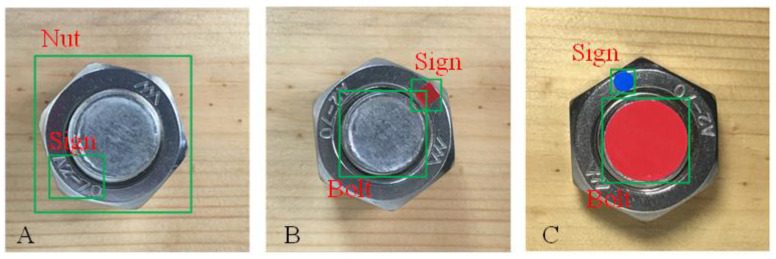
Collected images of the three schemes (**A**–**C**).

Image collection was carried out using a smart phone (pixel 3024 × 3024, resolution ratio 72 dpi) as the collecting device (Figure 2). The photographic distance was set at 8 to 10 cm. In order to ensure clear features, the area of the bolt pattern in each image obtained covered more than 25% of the whole image and flash and other methods can be used to ensure sufficient light. Each scheme collected 210 images in JPG format, in which 70% were used for training sets, while the remaining 30% were used for the testing sets. The color space of the picture was based on the default RGB. Then, the objects in the images were classified and named with a tool—LabelImg. The objects in scheme A were a bolt with the specification pattern “A2-70” and were labelled “Nut” and “Sign”, for which “A2-70” is the label indicating 304 stainless steel (Figure 3A). The objects in scheme B are the round feature on a bolt bottom and the rectangle on a nut, namely “Bolt” and “Sign”, respectively (Figure 3B). A rectangle sticker was attached on the nut as the feature in testing, while in practical production it would be sprayed with paint to improve manufacturing efficiency. The objects in scheme C are the round features on a bolt and a nut, namely “Bolt” and “Sign”, respectively (Figure 3C). The colors of the two round features were arbitrarily selected, but the colors of the two features must be different.

**Figure 4:**
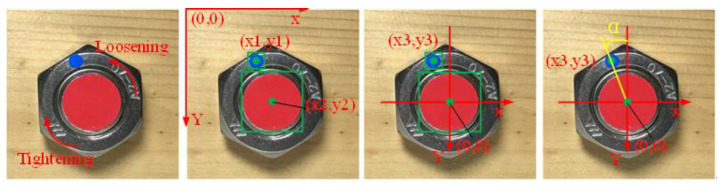
Rotation angle calculation process.

All of the objects were identified automatically, and the coordinates of center were generated after being trained using the SSD deep learning network. The calculation method of the loosening angle uses scheme C as an example, as shown in Figure 4. The tightening and loosening direction were determined prior to calculation. In an image identified by SSD, the origin of the coordinate was defaulted to the top left corner, and the center coordinates of the objects were $\left(x_{1},y_{1}\right)$ and $\left(x_{2},y_{2}\right)$, respectively. For the calculation, the origin of coordinate was transferred to the center of the bolt, turning the object’s coordinate on the nut into $\left(x_{3,}y_{3})=(x_{1}-x_{2},y_{1}-y_{2}\right)$. The angle of α is between $\left(x_{3},y_{3}\right)$ and the negative direction of the Y axis was in calculation angle, ranging from 0° to 360°. The calculation process is shown in Formula (1). In the actual operating environment, the initial angle of $\alpha_{0}$ was immediately recorded when a bolt was fastened. Subsequently, the angle of $\alpha_{i}$ was recorded when the bolt loosened. The looseness angle of a bolt can be expressed as *θ* = *α_i_* − *α*_0_.

(1) $$\begin{array}{c} \alpha =\{\begin{array}{c} 0,x_{3}=0,y_{3}<0\\ \arctan(\frac{x_{3}}{y_{3}}),x_{3}<0,y_{3}<0\\ 90,x_{3}<0,y_{3}=0\\ 90+\arctan(\frac{-y_{3}}{x_{3}}),x_{3}<0,y_{3}>0\\ 180,x_{3}=0,y_{3}>0\\ 180+\arctan(\frac{x_{3}}{y_{3}}),x_{3}>0,y_{3}>0\\ 270,x_{3}>0,y_{3}=0\\ 270+\arctan(\frac{-y_{3}}{x_{3}}),x_{3}>0,y_{3}<0 \end{array} \end{array}$$

### 2.2. Training Process

The deep learning network training was based on Single Shot MultiBox Detector (SSD) [23] algorithm under the pytorch framework using pycharm software. The SSD algorithm was part of one-stage methods. First, we conducted intensive sampling of various dimensions and aspect ratios uniformly in a single image. Then, the features were extracted using CNN to directly proceed to classification and regression. It obviously demonstrated the high efficiency of the SSD algorithm as the entire process could be realized in one stage.

Among the deep learning vision algorithms in the field of target detection, the SSD algorithm runs faster than the YOLO algorithm and the detection accuracy is more accurate than the Faster RCNN algorithm. Compared with the traditional machine vision algorithm, this algorithm is more robust and the feature setting is completely learned by the neural network. Besides, the learning features generally exceed the artificial features, and the hardware consumption cost of the algorithm is lower. Traditional machine vision algorithms are often complex to adjust parameters and difficult to use, so they are not suitable for beginners. SSD algorithm based on deep learning model only needs to understand the input and output, and basically can get relatively perfect results. In simple and fixed cases, traditional algorithms may be better than deep learning in computing resource consumption, while in complex and disturbing situations, they are not comparable to deep learning models.

**Figure 5:**
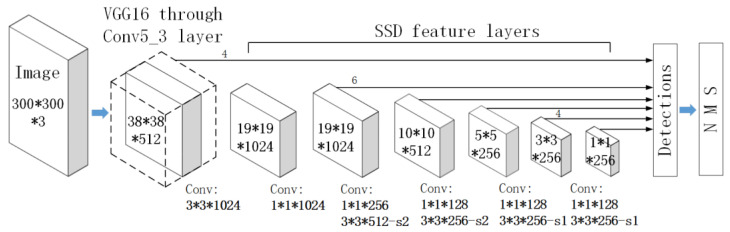
Single Shot MultiBox Detector (SSD) network structure.

**Figure 6:**
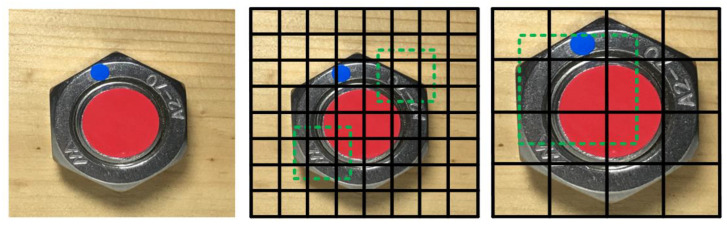
Features in multi-scales.

**Figure 7:**
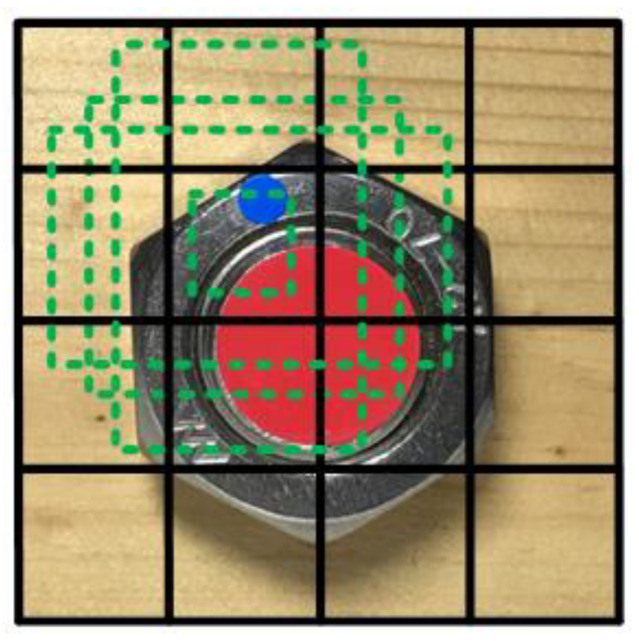
Prior boxes in a feature image.

The architecture of SSD is mainly divided into two parts. One is the deep convolution neural network located at the front end, which uses the image classification VGGNet-16 network without classification layer for the preliminary feature extraction of the target. The other part is the multi-scale feature detection network located at the back end, which is a group of cascaded CNN, to extract the features of the feature layer generated by the front-end network at different scales. The SSD framework is shown in Figure 5. There are three core contents of SSD algorithm.

(A) Images of multi-scale features were collected for detection. In CNN processing, the features were often large in the early stage, and the scale of the features was be reduced in the later stage through convolution and pooling. The features of large scales were used to detect small objects, while the features in small scales were used to detect large ones. Large-scale features could be divided into more units; nevertheless the prior boxes of each unit were relatively small (Figure 6).
(B) Convolution was utilized for detection. The SSD algorithm directly utilized convolution to extract the detection results from different feature images. It was simple to identify the features in the shape of m×n×p only processed with the convolution kernel in the shape of 3×3×p.
(C) Prior boxes were set in the training for detection. The SSD algorithm referred to the anchor idea from the Faster R-CNN setting for the prior boxes of different dimensions or aspect ratios on each unit. It predicted the bounding boxes according to these prior boxes, which simplified the feature training to some degree. Under normal circumstances, more than one prior box of distinguished dimensions and aspect ratios were set on each unit (Figure 7).

In the SSD training processing, it was essential to determine the specific prior boxes matching the ground truth in the trained image. The bounding boxes relative to the determined prior boxes predicted the feature. Two possible matching principles in the training processing were considered, as follows. The first was bipartite matching, where each ground truth box was matched to the source box with the best overlap. This was the matching approach used by the original Multibox, and it ensured that each ground truth box had exactly one matched source box. Per-prediction matching was also used for testing, which first used bipartite matching so that each ground truth box had one corresponding source box. It then matched the remaining source boxes to the most overlapped ground truth boxes if the overlap was higher than the threshold (e.g., 0.5). Unlike the bipartite matching, the per-prediction matching can generate several positive prior box matches for each ground truth. This allows the network to predict high confidences for multiple overlapping prior boxes rather than requiring it to always pick the best possible prior boxes [20].

### 2.3. Model Training Results

The training dataset of each scheme contained 210 images, where 70% were the training set and the remaining 30% were the testing set. The images were not pre-processed. In the training processing, the loss of model decreased with the increment of iteration. The identification accuracy reached a high level until the loss became stable. To improve the training rate, the experimental parameters were set as follows. Batch size was 4, learning rate decay factor was 0.94, optimizer was SGD (stochastic gradient descent), the initial learning rate was 0.001, and the training step was set as 10,000. After the 10,000-step training, the preliminary model generated was trained with 10,000 steps again with a learning rate of 0.0001. The results from the three schemes were compared to decide the optimum.

**Figure 8:**
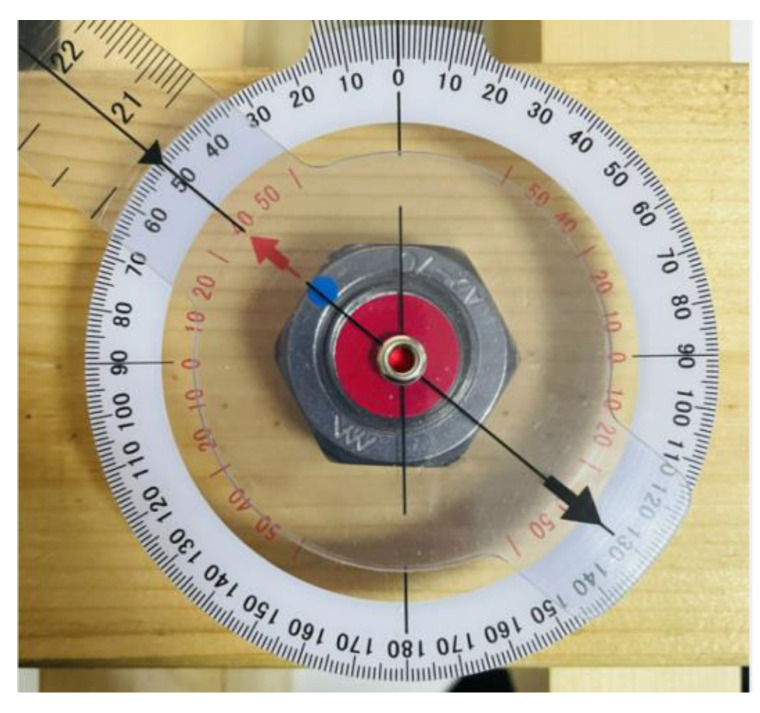
Setup of angle measurement.

In order to discover the accuracy of the three schemes, new images were collected for measurement. Images of a nut rotating 50°, 150°, 250°, and 350° were taken (Figure 8). Three images were collected at each angle. The camera needed to be facing the nut so that it covered at least 1/3 of the image. Afterwards, the rotation angle identification was conducted with the trained model. The results of the three schemes are presented in Table 1.

**Figure 9:**
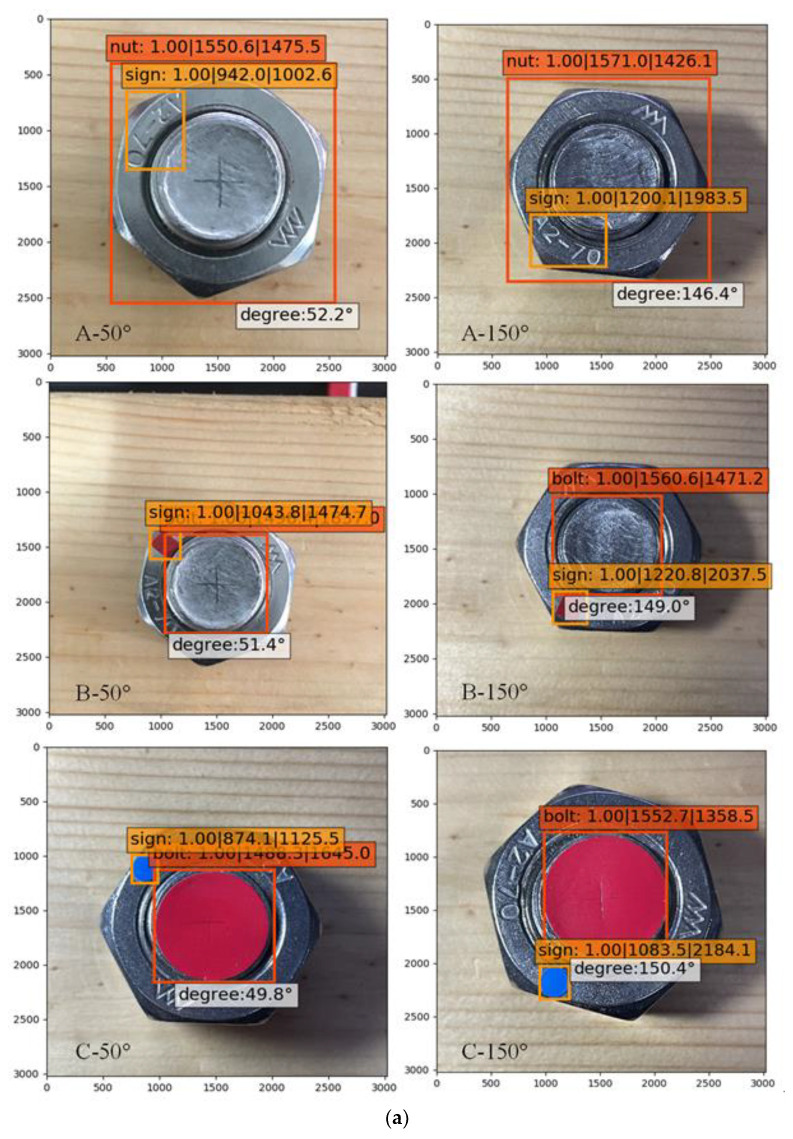

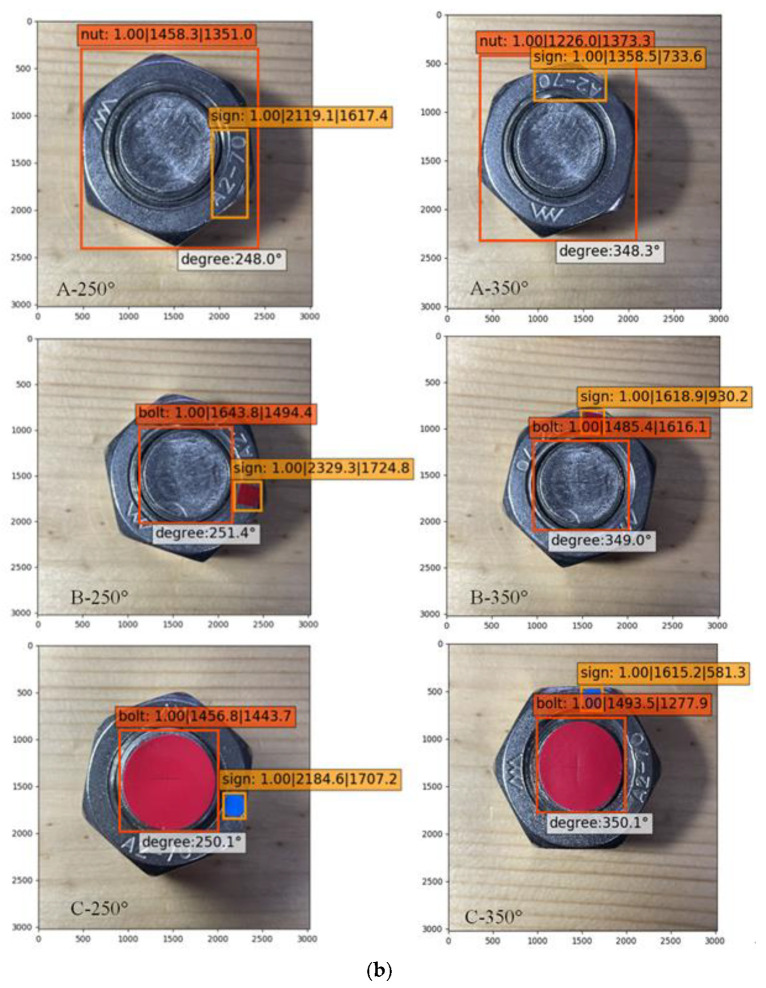
Best identification of rotation angles: (**a**) 50° and 150°; (**b**) 250° and 350°.

**Figure 10:**
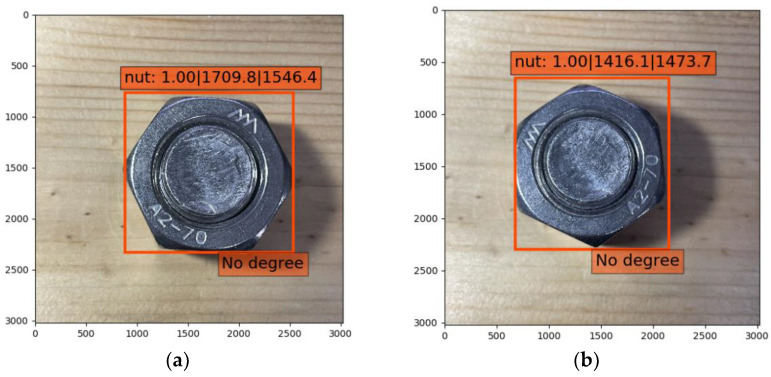
Identification results of scheme A: (**a**) initial state of a nut; (**b**) state of a nut after rotation.

The best recognition result of each model is shown in Figure 9. After training twice, all of the identification accuracies of the objects in the bolt images were 100%. After the calculation, the average angle errors were 2.65° in scheme A, 1.63° in scheme B, and 0.38° in scheme C, among which the smallest identification error was present in scheme C. In scheme A, on account of the small difference between the feature “Sign” and background, a large number of objects in the collecting and training could not be identified (Figure 10). It could be concluded that the definition of an image showed a tremendous influence on the identification. Furthermore, in scheme A, it was hard to determine the coordinate center of for identification, because the objects of an irregular shape are displayed using rectangular boxes with the SSD algorithm, which leads to large errors in the results. In scheme B, however, the same problem of measurement of for scheme A was not present. The coordination center perfectly matched the feature of “Bolt” for a round shape, but it did not work well when matching the rectangle feature of “Sign”. In addition, the object box changed with the feature “Sign” to sloping when the nut rotated. In scheme C, features are distinguished by red and blue colors, which helps the SSD algorithm to distinguish the target object during self-training and the identification accuracy of the rotation angle were improved. Therefore, considering the precision assessment of the schemes, scheme C, with its high accuracy and efficiency, was adopted to measure bolt looseness of the timber structure.

### 2.4. Impact of Lighting Condition

**Figure 11:**
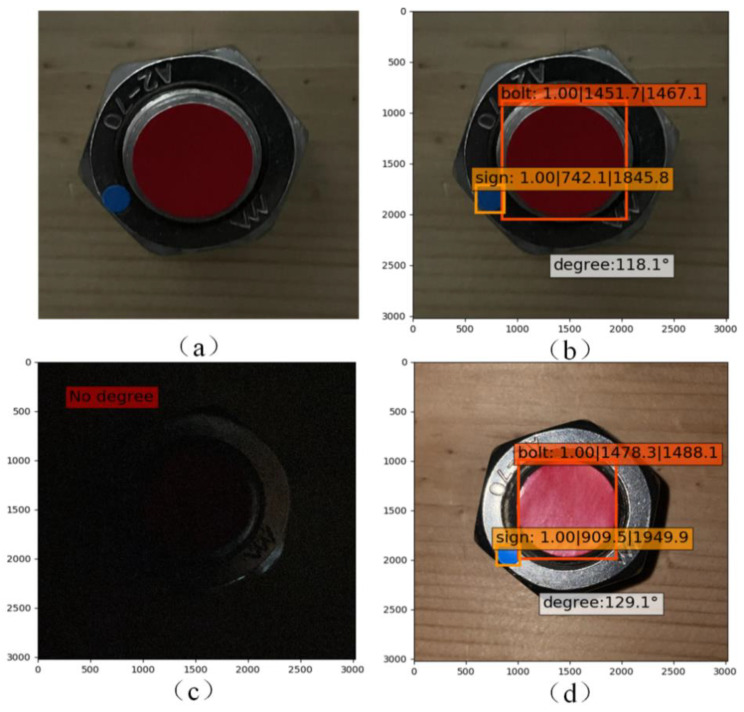
Verification results of lighting condition and shadow influence: (**a**) objects shot under weak lighting condition; (**b**) identification of the objects; (**c**) object shot under dark condition; (**d**) identification result of the object shot under camera flash.

Lighting condition was adjusted to ensure a good definition of the shot images in this study. However, in practical applications the real time monitoring will face the problem of different illumination intensity and shadow produced by lighting. To test the impact of them, the images were collected under relatively weak lighting and dark lighting conditions. Figure 11 respectively presents the verification results. It is observed that the training model generated from the objects collected under a clear condition still has high identification accuracy of the image collected under weak lighting. For the object which cannot be identified under dark lighting, it can also be accurately identified from the image shot under a camera flash. Using camera flash is a cost saving way to resolve the problem of weak lighting condition.

## 3. Results

### 3.1. Test for Precision of Minimum Identification Angle

**Figure 12:**
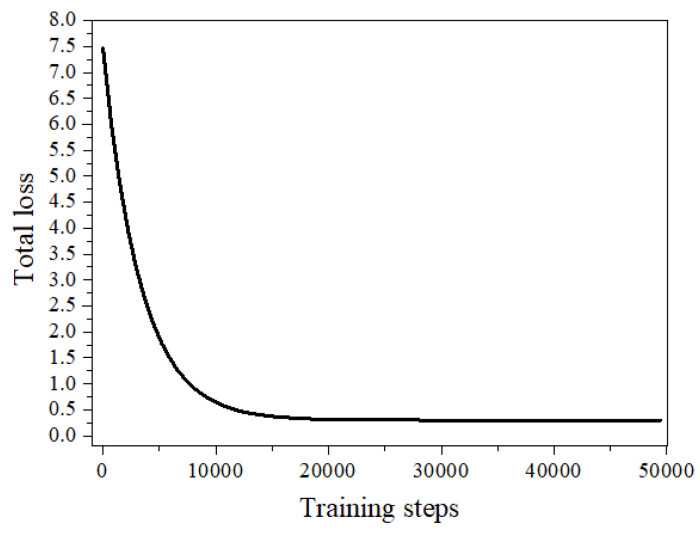
Loss decline curve.

In the bolted connection of a timber structure, the nut rotation angle is likely small when looseness occurs. The looseness becomes more obvious and serious as the nut rotates to create a larger angle, leading to a large possibility of structural failure. Hence, it is necessary to discover the bolt looseness early so as to prevent structural failure. To resolve the problem, this study carried out a test to identify the accurate measurement of the smallest identification angle. In order to improve the accuracy, 50,000 steps were trained again on the secondary training model of scheme C, with a learning rate of 0.00001. For the deep learning algorithm, the evaluation of the model was mainly based on parameters such as loss, average precision (AP), and mean AP (MAP). Loss refers to the errors between the predicted results and the actual results in the training sets, AP is the identification accuracy of each class, and MAP represents the average identification accuracy of all of the classes. There are usually many object categories identified by deep learning algorithms, but the model trained by scheme C in this study only identified two categories, namely “Sign” and “Bolt”. Both the identification accuracy and average identification accuracy of the class were 0.999. The loss decline curve of the third training is shown in Figure 12. At this time, the loss curve was almost in a stable state, indicating that the model had converged and the recognition effect of the model was close to stability.

**Figure 13:**
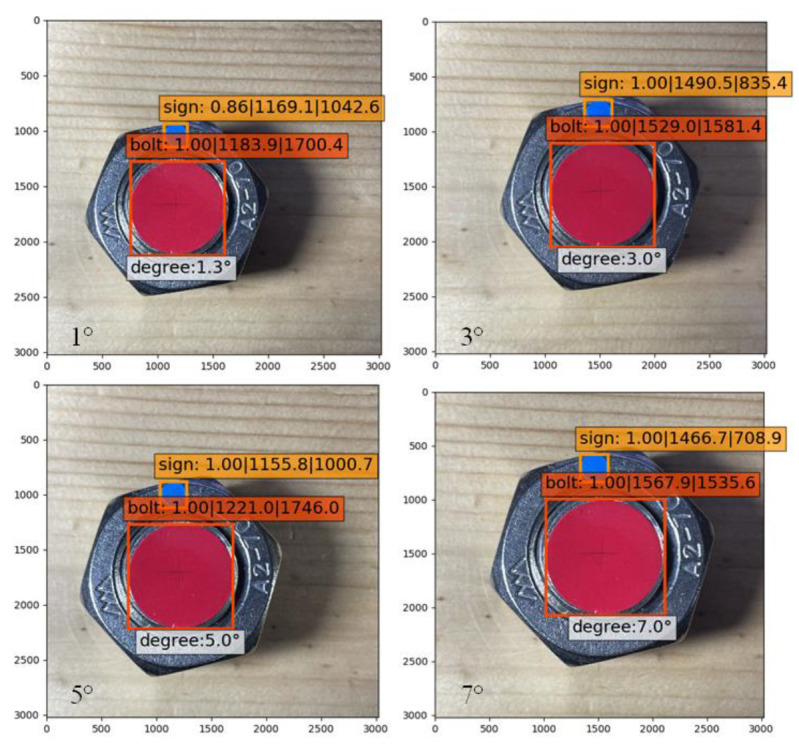
Best identification results at a tiny angle.

As the accuracy of the angle measuring instrument had a minimum of 1°, images of 1°, 3°, 5°, and 7° were measured and identified for testing. Three images were collected at each angle. Through the self-training model, the best results identified are shown in Figure 13.

The results at a small angle identification are shown in Table 2. The identification errors of the four angles were 0.37°, 0.1°, 0.07°, and 0.2°, respectively, and the overall average error was 0.185°. It was observed that increasing the training times of the model reduced the error of the angle identification to a certain extent. In addition, considering the errors given by manual measurement, this detection method could be considered to have a high identification precision, and the minimum identification angle reached 1°. It effectively detected bolt looseness at an early stage.

### 3.2. Effect of Multi-Bolt Identification

**Figure 14:**
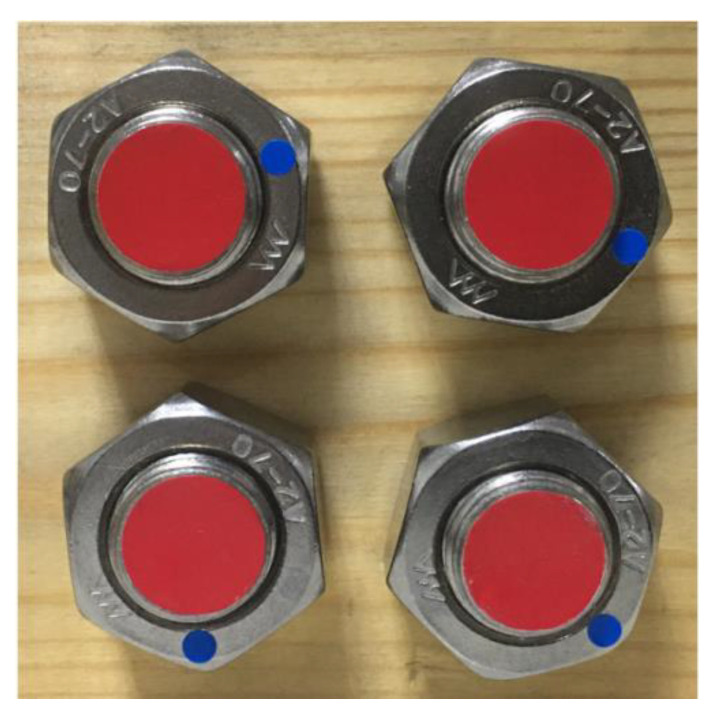
Four-bolted connection.

In practical working conditions, timber structure connections are fastened with more than one bolt than usual. It is relatively inefficient to detect only one bolt’s looseness once; on the contrary, the efficiency will be considerably increased if multiple bolts are detected simultaneously. It is of great importance to also train and test the multi-bolt looseness angles. In this study, a four-bolted connection was chosen as the experimental sample (Figure 14). Here, 210 pieces of images were collected with the same smart phone, of which the resolution was 3024 × 3024; 70% of them were used as the training set, and the remaining 30% were used as the testing set. The two categories of features, “Sign” and “Bolt” were trained with 10,000 steps, with learning rates of 0.001 and 0.0001 (20,000 steps in total), respectively.

**Figure 15:**
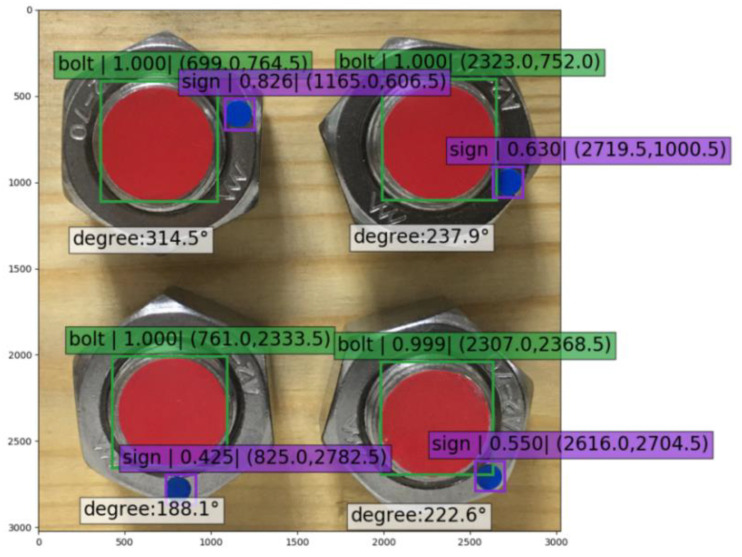
Identification results of a four-bolted connection.

When the angles of the four bolts were identified simultaneously in a single image, the identification accuracy of “Bolt” was still close to 100%, while that of “Sign” declined relatively (Figure 15). From the calculation of the testing sets, the average accuracy resulted in 65.7%. Compared with the identification of a single-bolted connection, the multi-bolted condition shrank the feature “Sign” displayed on a single image, leading to a training difficulty increase and identification accuracy decrease. Otherwise, according to the results, it was feasible to detect the looseness angle of a multi-bolted connection in a timber structure.

**Figure 16:**
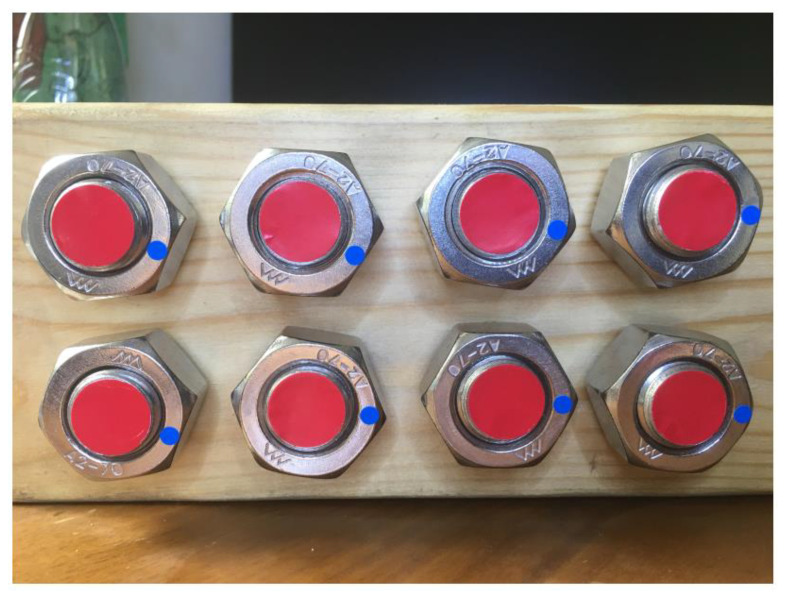
Eight-bolted connection.

**Figure 17:**
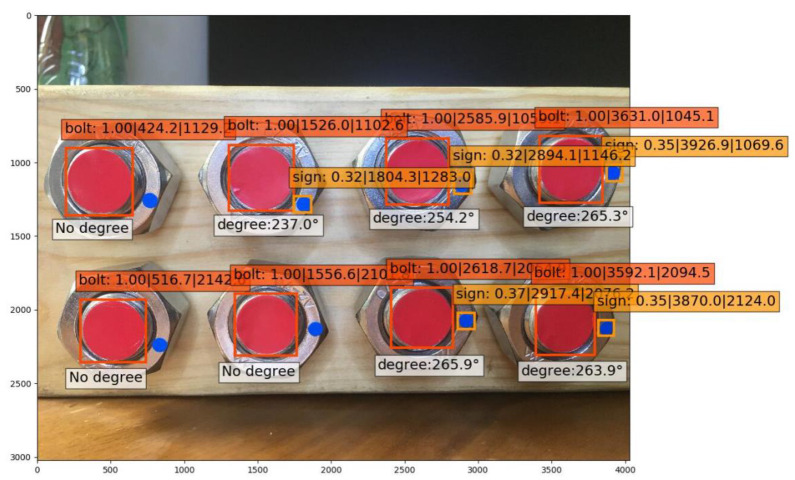
Identification results of an eight-bolted connection.

For the eight-bolted connection (Figure 16), which was trained with the same method and parameters as the four-bolt, the angle identification accuracy of the “Bolt” was close to 100%. However, only five features of “Sign” were identified, and the accuracy declined further to 35% (Figure 17).

**Figure 18:**
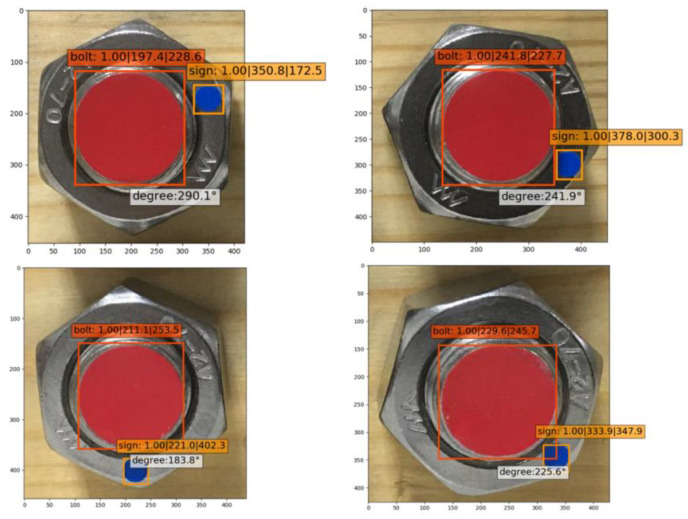
Secondary identification results of a multi-bolted connection.

**Figure 19:**
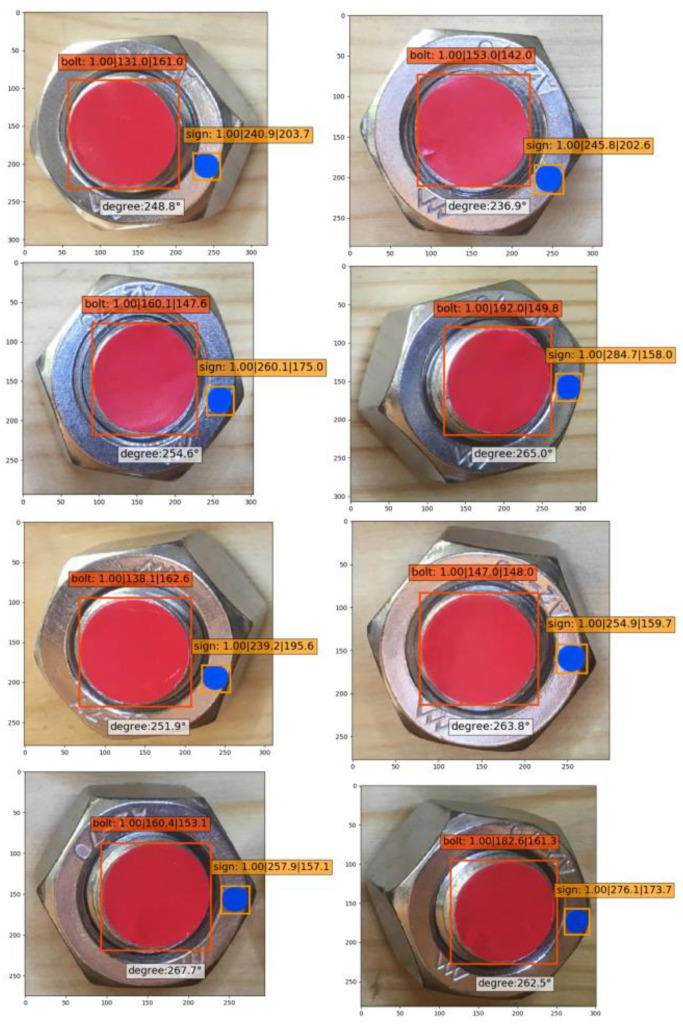
Identification results of an eight-bolted connection after cropping.

To solve the accuracy decline in identification of the small object “Sign” in one shooting detection of multi-objects, image cropping was adopted to conduct secondary identification (Figure 18). Based on the center coordinate of object “Bolt” determined from the first identification, each bolt connection was cropped into a single image for secondary identification. It is shown that the identification accuracy of “Sign” almost reaches 100%, which verifies the good feasibility of the study method on multi-object (Figure 19).

Therefore, though the identification accuracy for the small feature “Sign” with the SSD algorithm decreased with the increase in the amount of bolts, it can be corrected by secondary identification. In order to achieve better identification of small features, the vision algorithm could be optimized for ulterior uses. Meanwhile, to enable the identification of multi-bolted looseness angles, prior to model training, each bolt feature was extracted from a single image for angle identification. The testing method was the same as that on a single-bolted connection.

## 4. Discussion

The above sections verified the effectiveness of the small angle detection method for timber structure bolt looseness based on deep learning. Through a comparison of the three methods, an angle testing method with less errors and higher precision was developed. In the round feature identification of bolts and nuts, the angle error was an average of 0.38°, and the minimum identified angle reached 1°. The feasibility of the identification on multi-bolted connection was also ensured. Furthermore, the increase in the number of bolts leads to a decrease in recognition accuracy, which can be improved by improving the visual algorithm and dividing large pictures into small pictures. In this study, the camera was constantly facing the sample when all of the training images were collected, all of the tests resulted in good effectiveness, and the tests followed strict practical working conditions with a certain shooting cost. The pictures used in this paper can be taken under sufficient lighting conditions, such as using a flash to ensure the clarity of the picture. If in the actual environment, the lighting conditions cannot always ensure the clarity of the picture. Therefore, the lighting conditions and the shooting angle of the camera can be further studied as other influencing factors.

## 5. Conclusions

The detection method of small angle of loosening for timber structure bolts based on deep learning was studied. Three schemes were designed with training objects containing the specification pattern of an irregular shape on a nut, rectangle shape on a nut, and round shape on a bolt. Based on the pytorch deep learning frame, the image datasets were trained using the SSD algorithm to generate identification models. It was shown in the results that the rotation angle errors of scheme A, B, and C were 2.65°, 1.63°, and 0.38°, respectively. Scheme C, with the smallest errors, was used to further increase the training amount, which improved the identification accuracy and detected the identification effectiveness at small rotation angles. The results showed that using this method, the small angle error was an average of 0.185°, and the minimum identified angle reached 1°. Subsequently, the method for testing the looseness of a multi-bolted connection was implemented in order to simulate the practical working conditions. It demonstrated perfect feasibility on multi-bolted connection looseness. To summarize, this detection method for a small angle of loosening of timber structure bolts based on deep learning realized a low operating costing and obtained a high accuracy. Of great importance, it also provided good technical references for the detection of bolted connection looseness of a timber structure at an early stage.

## Figures and Tables

**Table 1:** Results of rotation angle (degree) identification.

	Scheme	A		B		C	
Rotation Angle		Angle Identified	Error	Angle Identified	Error	Angle Identified	Error
50		52.7	2.7	51.4	1.4	50.5	0.5
		52.2	2.2	52.1	2.1	50.7	0.7
		52.6	2.6	52.2	2.2	49.8	0.2
150		146.4	3.6	149.0	1.0	150.4	0.4
		146.2	3.8	148.7	1.3	149.2	0.8
		146.0	4.0	148.2	1.8	149.4	0.6
250		248.0	2.0	251.4	1.4	250.1	0.1
		247.9	2.1	251.9	1.9	249.4	0.6
		247.8	2.2	252.0	2.0	250.2	0.2
350		352.8	2.8	348.7	1.3	349.9	0.1
		348.3	1.7	349.0	1.0	350.1	0.1
		347.9	2.1	347.9	2.1	350.3	0.3
Average error			2.65		1.63		0.38

**Table 2:** Identification results at tiny angle (degree).

	Angle Measured	1	3	5	7
Sample					
①	Angle identified	1.3	3.2	5.1	6.5
	Error	0.3	0.2	0.1	0.5
②	Angle identified	0.6	3.0	5.0	7.1
	Error	0.4	0.0	0	0.1
③	Angle identified	1.4	2.9	4.9	7.0
	Error	0.4	0.1	0.1	0.0
Average error		0.37	0.1	0.07	0.2

## Data Availability

Not applicable.

## References

[1] Goodier J.N., Sweeney R.J. (1945). Loosening by vibration of threaded fastenings. Mech. Eng..

[2] Sauer J.A., Lemmon D.C., Lynn E.K. (1950). Bolts: How to prevent their loosening. Mach. Des..

[3] Bickford J. (2008). Other ways to control Preload. Introd. Des. Behav. Bolted Jt. Nongasketed Jt..

[4] Joshi S.G., Pathare R.G. (1984). Ultrasonic instrument for measuring bolt stress. Ultrasonics.

[5] Huo L., Wang F., Li H., Song G. (2017). A fractal contact theory based model for bolted connection looseness monitoring using piezoceramic transducers. Smart Mater. Struct..

[6] Xu C., Wu G., Du F., Zhu W., Mahdavi S.H. (2019). A Modified Time Reversal Method for Guided Wave Based Bolt Loosening Monitoring in a Lap Joint. J. Nondestruct. Eval..

[7] Zhao Z., Chen P., Zhang E., Lu G. (2019). Health Monitoring of Bolt Looseness in Timber Structures Using PZT-Enabled Time-Reversal Method. J. Sens..

[8] Zhang Y., Zhao X., Sun X., Su W., Xue Z. (2019). Bolt loosening detection based on audio classification. Adv. Struct. Eng..

[9] Wang F., Song G. (2020). Monitoring of multi-bolt connection looseness using a novel vibro-acoustic method. Nonlinear Dyn..

[10] Wang C., Xu X. (2016). An extended phantom node method study of crack propagation of composites under fatigue loading. Compos. Struct..

[11] Wang C. (2020). Transverse crack evolution modeling of cross-ply laminates with a single layer of phantom node intraply elements for identically-oriented ply groups. Compos. Struct..

[12] Park J.H., Huynh T.C., Choi S.H., Kim J.T. (2015). Vision-based technique for bolt-loosening detection in wind turbine tower. Wind Struct. Int. J..

[13] Nguyen T.C., Huynh T.C., Ryu J.Y., Park J.H., Kim J.T., Yu T., Gyekenyes A.L., Shull P.J., Wu H.F. (2016). Bolt-loosening identification of bolt connections by vision image-based technique. Nondestructive Characterization and Monitoring of Advanced Materials, Aerospace, and Civil Infrastructure 2016.

[14] Cha Y.-J., You K., Choi W. (2016). Vision-based detection of loosened bolts using the Hough transform and support vector machines. Autom. Constr..

[15] Shi J., Li Z., Zhu T., Wang D., Ni C. (2020). Defect Detection of Industry Wood Veneer Based on NAS and Multi-Channel Mask R-CNN. Sensors.

[16] Krizhevsky A., Sutskever I., Hinton G.E. (2017). ImageNet Classification with Deep Convolutional Neural Networks. Commun. Acm.

[17] Simonyan K., Zisserman A. (2014). Very Deep Convolutional Networks for Large-Scale Image Recognition. arXiv.

[18] Szegedy C., Vanhoucke V., Ioffe S., Shlens J., Wojna Z. Rethinking the Inception Architecture for Computer Vision. Proceedings of the 2016 IEEE Conference on Computer Vision and Pattern Recognition.

[19] Girshick R., Donahue J., Darrell T., Malik J. Rich feature hierarchies for accurate object detection and semantic segmentation. Proceedings of the 2014 IEEE Conference on Computer Vision and Pattern Recognition.

[20] Girshick R. Fast R-CNN. Proceedings of the 2015 IEEE International Conference on Computer Vision.

[21] Ren S., He K., Girshick R., Sun J. (2017). Faster R-CNN: Towards Real-Time Object Detection with Region Proposal Networks. IEEE Trans. Pattern Anal. Mach. Intell..

[22] Redmon J., Divvala S., Girshick R., Farhadi A. You Only Look Once: Unified, Real-Time Object Detection. Proceedings of the 2016 IEEE Conference on Computer Vision and Pattern Recognition.

[23] Liu W., Anguelov D., Erhan D., Szegedy C., Reed S., Fu C.-Y., Berg A.C., Leibe B., Matas J., Sebe N., Welling M. (2016). SSD: Single Shot MultiBox Detector. European Conference on Computer Vision.

[24] Sun J., Xie Y., Cheng X. (2019). A Fast Bolt-Loosening Detection Method of Running Train’s Key Components Based on Binocular Vision. IEEE Access.

[25] Thanh-Canh H., Park J.-H., Jung H.-J., Kim J.-T. (2019). Quasi-autonomous bolt-loosening detection method using vision-based deep learning and image processing. Autom. Constr..

[26] Zhao X., Zhang Y., Wang N. (2019). Bolt loosening angle detection technology using deep learning. Struct. Control. Health Monit..

[27] Zhang Y., Sun X., Loh K.J., Su W., Xue Z., Zhao X. (2020). Autonomous bolt loosening detection using deep learning. Struct. Health Monit. Int. J..

[28] Junker G.H. (1972). Criteria for self loosening of fasteners under vibration. Aircr. Eng. Aerosp. Technol..

[29] Wi J.-H., Ahn H.-J., Lee K.-H., Lee C.-H. (2019). Self-Loosening Characteristics of Three-Dimensional Printed Bolted Joints. 3d Print. Addit. Manuf..

